# Hidden in Plain Pain: Diagnosing Ischemic Enteritis Through Surgical Intervention

**DOI:** 10.1002/ccr3.72927

**Published:** 2026-06-17

**Authors:** Aya Obata, Takaaki Kobayashi, Rui Ouchi, Akihito Yoshida

**Affiliations:** ^1^ Department of General Internal Medicine Kameda Medical Center Kamogawa Chiba Japan; ^2^ Department of General Internal Medicine Institute of Science Tokyo Bunkyo City Tokyo Japan; ^3^ Department of Internal Medicine University of Kentucky Lexington Kentucky USA

**Keywords:** chronic abdominal pain, ischemic enteritis, surgical intervention, vascular risk factors

## Abstract

An elderly woman with vascular risk factors presented with postprandial abdominal pain. CT showed ileal edema with the target sign but no vascular occlusion. Surgical resection confirmed ischemic enteritis. This case highlights a rare cause of chronic abdominal pain.

## Key Clinical Message

Ischemic enteritis should be considered in elderly patients with recurrent abdominal pain and vascular risk factors. We should recognize that surgical intervention can serve both diagnostic and therapeutic purposes in cases where ischemic enteritis is suspected but is difficult to confirm.

An 89‐year‐old woman with a history of myocardial infarction, type 2 diabetes mellitus, and dyslipidemia presented with a 3‐month history of progressively worsening postprandial abdominal pain. She had previously been hospitalized twice at another facility, where her symptoms improved temporarily with conservative management but recurred after resuming oral intake. On her third admission, she remained hemodynamically stable, but continued to report significant pain following meals. Physical examination was unremarkable, and laboratory findings were within normal limits. Given her cardiovascular risk factors, intestinal ischemia was suspected. Contrast‐enhanced abdominal computed tomography (CT) revealed localized wall thickening and edema of a segment of the distal ileum with mild proximal small bowel dilation (Figure 1, yellow arrows). Mesenteric angiography demonstrated no evidence of significant stenosis, thromboembolism, or arterial occlusion (Figure 2). Double‐balloon enteroscopy was considered to allow direct visualization and possible biopsy of the affected segment, but this was deferred due to concerns about procedural risk in the context of her advanced age and cardiac history. Despite continued conservative management, her postprandial pain persisted and interfered with nutritional intake. On hospital day 28, she underwent laparoscopic‐assisted partial small bowel resection. Intraoperatively, a thickened segment of ileum approximately 40 cm proximal to the ileocecal valve, corresponding to the lesion identified on CT imaging, was observed with mild upstream dilation. The affected bowel segment was exteriorized through an extended umbilical incision and resected with approximately 3 cm margins on both the proximal and distal sides. Reconstruction was performed using a stapled side‐to‐side functional end‐to‐end anastomosis. The resected specimen demonstrated a 4‐cm segment of circumferential stenosis with focal discoloration and firm underlying tissue (Figure 3). Histopathological analysis of the surgical specimen revealed circumferential ulceration, transmural fibrosis, and chronic inflammation, without signs of malignancy, vasculitis, or thrombi—findings consistent with ischemic enteritis (Figure 4). The patient resumed oral intake on postoperative day 2 and experienced complete resolution of postprandial abdominal pain, with no recurrence during follow‐up.

**FIGURE 1:**
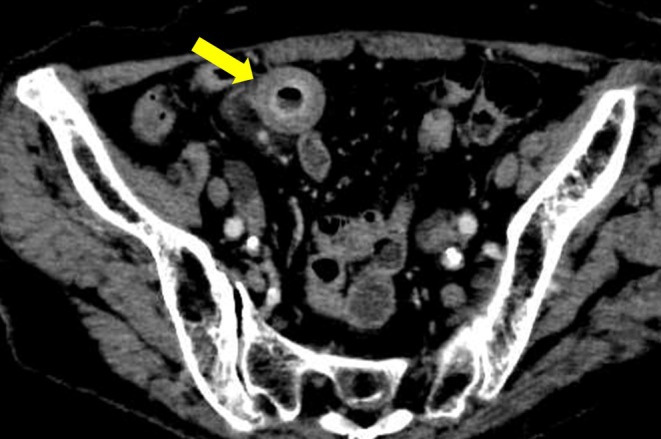
Contrast‐enhanced CT revealed edema around a segment of the ileum, known as the “target sign”, and mild proximal bowel dilation.

**FIGURE 2:**
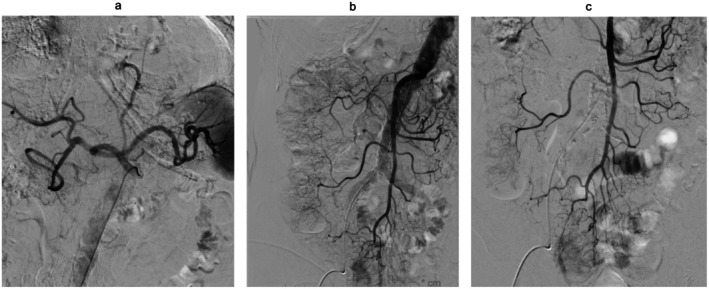
An angiogram identified no stenosis or thrombosis in the corresponding blood vessels. (a) Celiac artery, (b) superior mesenteric artery, and (c) inferior mesenteric artery.

**FIGURE 3:**
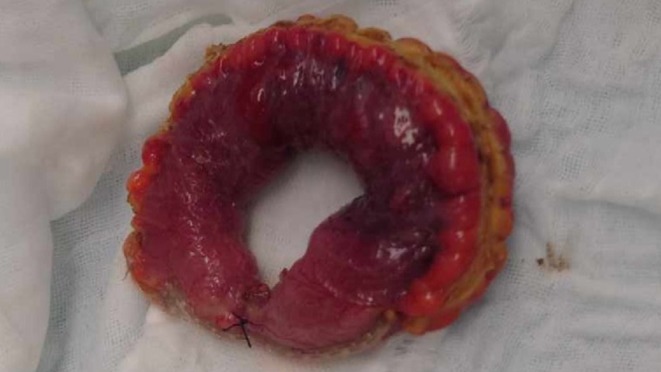
The resected bowel wall of a 4 cm segment of the ileum showed focal discoloration with firm underlying tissue.

**FIGURE 4:**
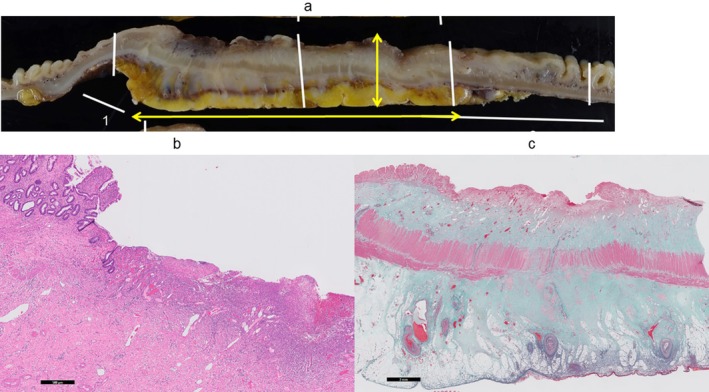
Histological examination of the biopsy specimens revealed no findings suggestive of a tumor or vascular lesions. (a) A portion of the resected small intestine showed marked wall thickening. There was circumferential bowel stenosis with proximal dilatation. (b) HE staining revealed ulcers and hemosiderin deposits. (c) Masson's trichrome staining showed destruction of the muscular layer and replacement by collagen fibers within adipose tissue.

Ischemic enteritis is a rare but important cause of chronic small bowel symptoms, typically resulting from insufficient arterial perfusion without major vessel occlusion. Unlike the more commonly recognized colonic ischemia, small bowel involvement is uncommon due to the extensive collateral circulation of the mesenteric vasculature [1]. Risk factors for ischemic enteritis include advanced age, diabetes mellitus, cardiovascular disease, and conditions associated with low‐flow states [2]. Clinical presentation may mimic chronic mesenteric ischemia, with postprandial pain being the hallmark symptom. In our case, repeated symptom recurrence upon refeeding, along with known atherosclerotic disease, prompted suspicion. Diagnosis is often delayed due to nonspecific clinical and radiologic findings. CT imaging may show bowel wall thickening, submucosal edema, and upstream dilation, but these features overlap with other enteropathies. Angiographic studies are valuable in ruling out large vessel occlusion, though microvascular ischemia may still be present. When endoscopic evaluation is not feasible, surgical resection may serve both diagnostic and therapeutic roles. Treatment options vary depending on the chronicity and extent of the disease. Endoscopic balloon dilation has been reported for short strictures, though high recurrence rates limit its long‐term efficacy. In most cases with fibrotic stenosis, surgery remains the definitive approach [3]. This case highlights the importance of maintaining a high index of suspicion for ischemic enteritis in elderly patients with recurrent abdominal pain and vascular comorbidities, particularly when symptoms fail to improve with conservative measures.

## Author Contributions

**Aya Obata:** writing – original draft. **Takaaki Kobayashi:** supervision. **Rui Ouchi:** writing – original draft. **Akihito Yoshida:** writing – review and editing.

## Funding

The authors have nothing to report.

## Consent

Written consent was obtained from the patient and is available upon request.

## Conflicts of Interest

The authors declare no conflicts of interest.

## Data Availability

The data that support the findings of this study are available on request from the corresponding author. The data are not publicly available due to privacy or ethical restrictions.
